# Simultaneous characterization of poly(acrylic acid) and polysaccharide polymers and copolymers

**DOI:** 10.1002/ansa.202000044

**Published:** 2020-06-05

**Authors:** Ruben Epping, Ulrich Panne, Wolf Hiller, Till Gruendling, Bastiaan Staal, Christiane Lang, Alexandros Lamprou, Jana Falkenhagen

**Affiliations:** ^1^ Bundesanstalt für Materialforschung und –prüfung (BAM) Berlin Germany; ^2^ Chemistry Department Humboldt Universität zu Berlin Berlin Germany; ^3^ Faculty of Chemistry and Chemical Biology TU Dortmund Dortmund Germany; ^4^ BASF SE Ludwigshafen am Rhein Germany; ^5^ Innovation Campus Asia Pacific BASF Shanghai China

**Keywords:** 2D chromatography, grafting, LC‐MS, renewable copolymers, size exclusion chromatography

## Abstract

Copolymer products that result from grafting acrylic acid and other hydrophilic monomers onto polysaccharides have recently gained significant interest in research and industry. Originating from renewable sources, these biodegradable, low toxicity, and polar copolymer products exhibit potential to replace polymers from fossil sources in several applications and industries. The methods usually employed to characterize these copolymers are, however, quite limited, especially for the measurement of bulk properties. With more sophisticated applications, for example, in pharmaceutics requiring a more detailed analysis of the chemical structure, we describe a new approach for this kind of complex polymers. Our approach utilizes chromatography in combination with several detection methods to separate and characterize reaction products of the copolymerization of acrylic acid and chemically hydrolyzed starch. These samples consisted of a mixture of homopolymer poly (acrylic acid), homopolymer hydrolyzed starch, and – in a lower amount – the formed copolymers. Several chromatographic methods exist that are capable of characterizing either poly (acrylic acid) or hydrolyzed starch. In contrast, our approach offers simultaneous characterization of both polymers. The combination of LC and UV/RI offered insight into the composition and copolymer content of the samples. Size exclusion chromatography experiments revealed the molar mass distribution of homopolymers and copolymers. FTIR investigations confirmed the formation of copolymers while ESI‐MS gave more details on the end groups of hydrolyzed starches and poly (acrylic acids). Evidence of copolymer structures was obtained through NMR measurements. Finally, two‐dimensional chromatography led to the separation of the copolymers from both homopolymers as well as the additional separation of sodium clusters. The methods described in this work are a powerful toolset to characterize copolymerization products of hydrolyzed starch and poly(acrylic acid). Together, our approach successfully correlates the physicochemical properties of such complex mixtures with their actual composition.

**Abbreviations**
AA: acrylic acid
HS: hydrolyzed starch
LCCC: liquid chromatography under critical conditions
MMD: molar mass distribution
PAA: poly(acrylic acid)
PS: polysaccharide
RI: refractive Index
SEC: size exclusion chromatography

## 1 INTRODUCTION

The possibility to use natural instead of fossil resources to produce polymeric materials has attracted growing interest. Poly(acrylic acid) (PAA) and polysaccharides are both used widely in various applications.^1^, ^2^, ^3^, ^4^, ^5^ Due to the large number of hydrophilic groups in their respective structures they are polar, water‐soluble polymers.

The biocompatible, biodegradable, and low toxicity copolymers between polysaccharides and PAA make them suitable for various applications, including as food additives, pharmaceuticals, agriculture chemicals, and in consumer products.^6^, ^7^, ^8^, ^9^, ^10^ Prominent uses of sophisticated polymer architectures include superabsorbents^11^ and drug delivery systems.^12^, ^13^ There are several ways to produce these copolymer products, most of which are based on grafting a monomer onto a polysaccharides backbone.^6^, ^14^, ^15^

The characterization of these industrially relevant polymers is poorly documented in literature. Simple analysis of the copolymerization reaction, after several washing and precipitation steps, is often described.^6^, ^14^ Moreover, the reaction yield is typically determined by gravimetric analysis of the product. Further analyses by FT‐IR, X‐ray diffraction, AFM, SEM, and Raman and NMR spectroscopies are also reported.^11^, ^16^, ^17^, ^18^ The kinetics and mechanism of the grafting process itself are not well understood.

Size exclusion chromatography (SEC) is the only reported chromatographic characterization method for these types of copolymers thus far.^19^ There do, however, exist methods that can characterize one of PAA or polysaccharides. Methods for the chromatographic separation and characterization of reaction products containing both homopolymers simultaneously are not known. Correspondingly, simultaneous quantification of the desired copolymer products in a mixture of their homopolymers is beyond current capabilities.

For other types of amphiphilic and water soluble graft copolymers, for example, with a PEG backbone, some chromatographic analyses are reported.^20^, ^21^, ^22^, ^23^, ^24^, ^25^ SEC,^26^ (gradient) liquid adsorption chromatography (LAC), and the less common liquid chromatography under critical conditions (LCCC)^22^ have been utilized. Graft copolymers were separated selectively with regards to molar masses, chemical composition, or functionality. By combining two of these chromatographic dimensions, a more sophisticated analysis could be achieved. Combining chromatographic dimensions comes with several challenges for characterization of copolymers solubilized in aqueous mobile phases. These challenges include the fact that (a) the number of available stationary phases is very limited, (b) ionic interactions that depend on mobile phase pH can occur, (c) salts present can cause problems for detection, and (d) mobile phases of dimensions in 2D‐chromatography can be incompatible and accurate calibration can be problematic. Complex purification strategies for reaction mixtures are often necessary before chromatographic analysis.^24^, ^27^

In this work, the completely soluble products of a copolymerization reaction between acrylic acid (AA) and hydrolyzed starch (HS) as polysaccharide were characterized with various analytical methods. We focused on the simultaneous characterization of a reaction mixture that contained both homopolymers besides the copolymer.

**FIGURE 1:**
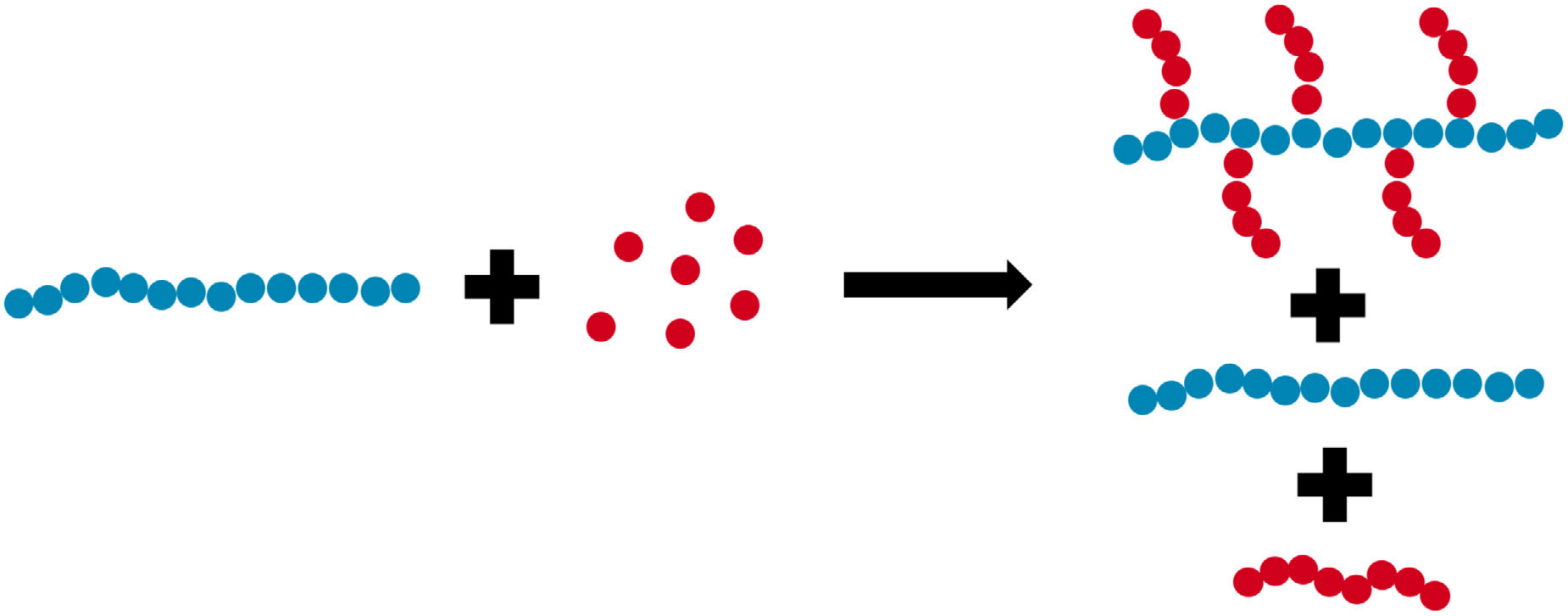
Grafting reaction principle

The synthesis was carried out as a free‐radical polymerization and resulted in a mixture of the copolymerization product alongside residual homopolymers. The copolymerization product is presumed to be a graft‐like structure of PAA grafted onto HS, most likely in positions C1, C2, or C6 of HS. Another possibility is an alcohol‐alcohol dehydration, resulting in the formation of an ether group. The principle of a graft reaction is shown in Figure 1. Alongside the formation of the product, it is expected that some amount of unreacted HS (blue) remains and that homopolymer PAA (red) will be formed.

**SCHEME 1:**
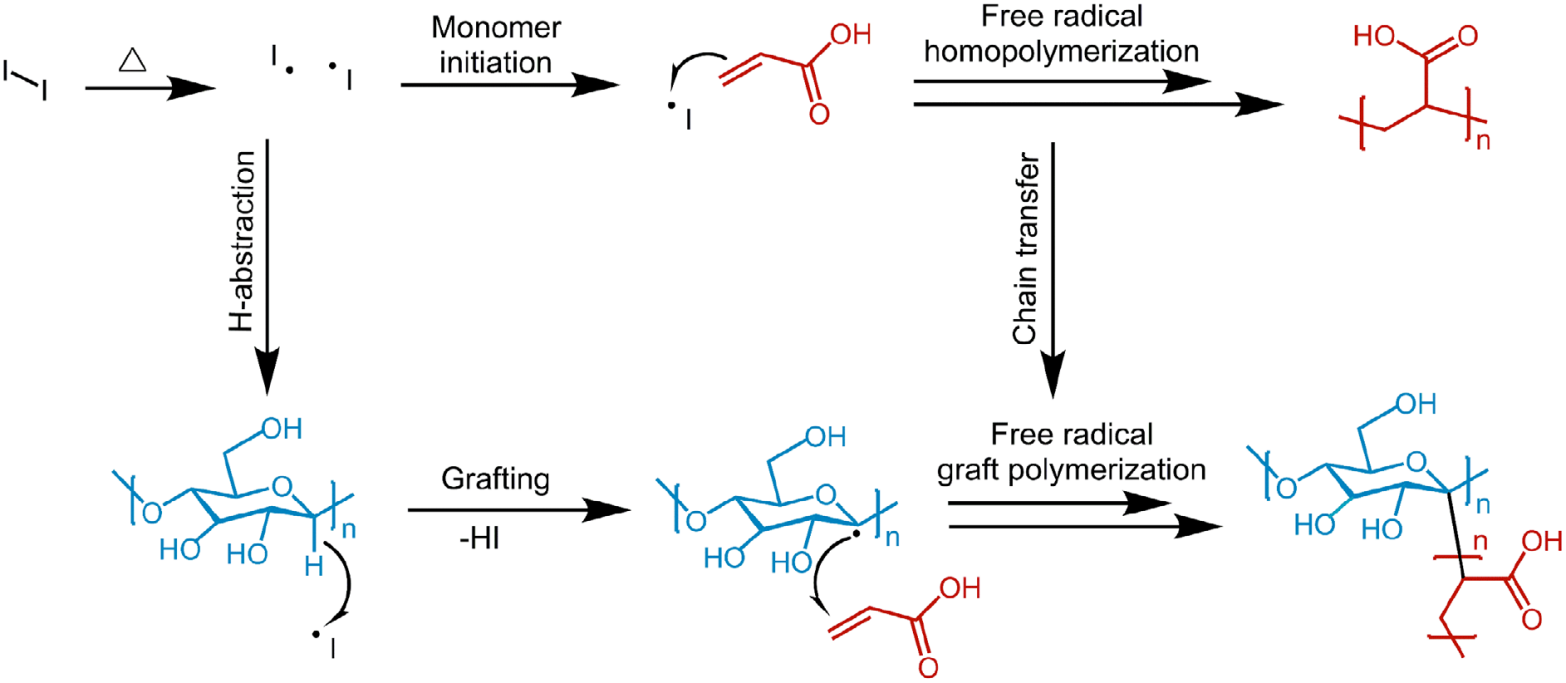
Possible reaction pathway of the copolymer formation. I , initiator

The proposed reaction scheme of the investigated synthesis, derived from literature known mechanisms,^21^, ^28^ is shown in Scheme 1. The exact compound structures and the reaction yield were to be determined. Due to the nature of the applied reactants, a distribution of end‐groups (eg, due to oxidative processes) is expected.

Overall, 11 samples were analyzed. These included our PAA samples with mean molar masses of 1250, 2500, 8000, and 15 000 g/mol, HS, and four copolymer samples with varying PAA and HS amounts or expected different length of PAA chains, respectively. Differences were achieved by altering the ratio of starting material and the reaction conditions. All assigned sample names and their respective compositions are summarized in Table 1.

**TABLE 1:** Characterized samples and their composition

Sample	Composition
PAA	Poly (acrylic acid) with known molar mass distribution
HS	Hydrolyzed starch
AA‐co‐HS 20:80	Copolymerization product with a ratio of starting materials AA:HS = 20:80
AA‐co‐HS 50:50 I	Copolymerization product with a ratio of starting materials AA:HS = 50:50
AA‐co‐HS 50:50 II	Copolymerization product with expected increased length of PAA chains in respect to AA‐co‐PS 50:50 I
AA‐co‐HS 50:50 III	Copolymerization product with further expected increased length of PAA chains

## 2 MATERIALS AND METHODS

### 2.1 Materials

AA, PAA with different molar masses (M_p_ 1250, 2500, 8000, and 15 000 g/mol) were purchased from commercial suppliers (PSS GmbH). Hydrolyzed starch (HS) was produced by chemically hydrolyzing starch. Copolymerization experiments were carried out with HS and AA. Table 1 lists all samples analyzed in this work.

### 2.2 Chromatography

For the chromatographic separations, three systems were employed. A UPLC system (Waters) equipped with an evaporative light scattering detector (ELSD) and an electrospray mass spectrometer (ESI‐TOF‐MS, QTOF Ultima, Waters, Micromass). Second, a 1260 Infinity HPLC system (Agilent Technologies) coupled to a variable wavelength UV‐detector and a refractive index (RI) detector. For 2D experiments, a 1260 Infinity system was combined with a 1200 Infinity system (Agilent Technologies) equipped with an ELSD (1290 Infinity II). Measurements were carried out on three different stationary phases.

For analyses on a Jordigel DVB Polyamine (500 Å, 250 × 4.6 mm, 5 µm particle size), the column temperature was set to 35°C with an eluent of bidest. Water containing 0.1% v/v of formic acid with a pH of 2. 10 µL of a 2 mg/mL solution was injected. The flow rate was 1 and 2 mL/min for 2D chromatography.

Measurements on a YMC‐Triart C18 ExRS (8 Å, 150 × 2.1 mm, 1.9 µm particle size) were carried out with an eluent of 85:15% v/v of water and acetonitrile (ACN) with 0.1% v/v formic acid added. For 2D experiments, a gradient from 10% to 30% ACN was used. The flow rate was 0.25 mL/min for isocratic and 0.05 mL/min for gradient experiments, the temperature was kept at 25°C. Two microliters of a 2 mg/mL solution were injected.

The size exclusion chromatography (SEC) measurements were performed with two PSS Suprema columns (300 × 8 mm, 10 µm particle size) of 100 and 1000 Å pore size, respectively. Water containing 0.1% v/v formic acid was used as eluent. The flow rate was 1 mL/min. Twenty microliters of a 2 mg/mL solution were injected at a column temperature of 35°C. Calibration was performed using PAA and Pullulan standards.

For all LC measurements, the samples were dissolved in a solvent resembling the eluent composition at the starting point of the gradient. UPLC/MS and HPLC grade solvents (Sigma–Aldrich) were used. 2D experiments used a Rheodyne six‐port switching valve with two 100 µL loops. Data were processed with WinGPC Unity (PSS), MassLynx 4.1 (Waters), and Origin 2019. An overview of which columns were used with which instrument and eluent is given in Table 2.

**TABLE 2:** Overview of Instruments, columns and eluents used in this work

Instrument	Column	Eluent
ACQUITY UPLC System (Waters) with ELSD and ESI‐TOF‐MS QTOF Ultima (Waters, Micromass)	YMC‐Triart C18 ExRS (8 Å, 150 × 2.1 mm, 1.9 µm particle size) at 25°C with injection of 2µL of a 2 mg/mL solution	85:15% v/v of H_2_O:ACN with 0.1 v/v % formic acid at 0.25 mL/min
1260 Infinity HPLC system coupled to a variable wavelength UV‐ and a RI‐detector (Agilent Technologies)	DVB Polyamine (500 Å, 250 × 4.6 mm, 5 µm particle size) at 35°C with injection of 10 µL of a 2 mg/mL solution	H_2_O with 0.1% v/v of formic acid at pH 2 at 1 mL/min
	Two PSS Suprema columns (300 × 8 mm, 10 µm particle size) of 100 and 1000 Å at 35°C with injection of 20 µL of a 2 mg/mL solution	H_2_O with 0.1% v/v of formic acid at 1 mL/min
1260 Infinity system combined with 1200 Infinity system (Agilent Technologies) with ELSD (1290 Infinity II) through Rheodyne six‐port switching valve with two 100 µL loops	YMC‐Triart C18 ExRS from at 25°C with injection of 20µL of a 20 mg/mL solution Plus DVB Polyamine at 35°	10 to 30% CAN at 0.05 mL/min in first dimension 2 mL/min in second dimension

### 2.3 Mass spectrometry

A Q‐TOF Ultima ESI‐TOF mass spectrometer (Micromass) running at 3 kV capillary voltage, a source temperature of 120°C and a desolvation temperature of 500°C was used for all measurements. The mass spectrometer was operated in the positive ion mode. A solution of 0.1 mg/L sodium trifluoroacetate was added to the eluent from the chromatography via a mixing T‐piece at a flowrate of 1 µL/min to improve ionization. Data were processed using MassLynx 4.1 (Waters).

### 2.4 FT‐IR spectroscopy

FT‐IR data were recorded on a Nicolet Nexus 8700 spectrometer (Thermo Electron). The spectra of samples deposited and dried on a germanium disc, were recorded from 4000 to 400 cm^−1^ in ATR mode. Sixty‐four scans were recorded for each sample. Data were processed with OMNIC software (Thermo Electron). The spectral background was measured before each sample and subtracted.

### 2.5 NMR spectroscopy

^1^H NMR measurements were obtained with a 600 MHz NMR spectrometer AVANCE‐III HD (Bruker), equipped with a 5 mm helium cooled BBFO cryoprobe. All NMR samples were dissolved in 0.5 mL D_2_O. The pulse sequence used 30° pulses of 3.9 µs. Sixteen scans were accumulated by using a relaxation delay of 6 s and an acquisition time of 2.7 s (corresponding to 64 kB for the FID and 256 kB for the Fourier transformation).

## 3 RESULTS AND DISCUSSION

### 3.1 UV spectroscopy

**FIGURE 2:**
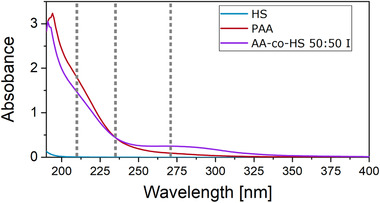
UV absorption spectra of both homopolymers and one of the copolymer samples. The wavelengths used for UV detection are highlighted by grey dotted lines (compare to Figure 3)

Figure 2 shows the UV spectra in the observed wavelength region of HS, PAA, and AA‐co‐HS 50:50 I. HS shows almost no absorption at any wavelength. The small absorption at lower wavelengths can be attributed to the absorption of the glass cuvette. PAA shows strong absorption at wavelengths below 235 nm and weaker absorption above 235 nm.

Since the copolymer sample is expected to contain significant amounts of homopolymer HS and PAA, the absorption spectra are similar to PAA. However, it appears that some amount of PAA reacted and a decrease in absorption intensity below 235 nm is observed. In return, an increase in absorption can be seen between 235 nm and 350 nm. Three wavelengths of 210, 235, and 270 nm (Figure 2) were recorded during the chromatographic experiments. Although 210 nm should indicate only PAA, the sensitivity to PAA should decrease over 235 nm to 270 nm, while the sensitivity toward the copolymer should increase.

### 3.2 LC on polyamine phase

**FIGURE 3:**
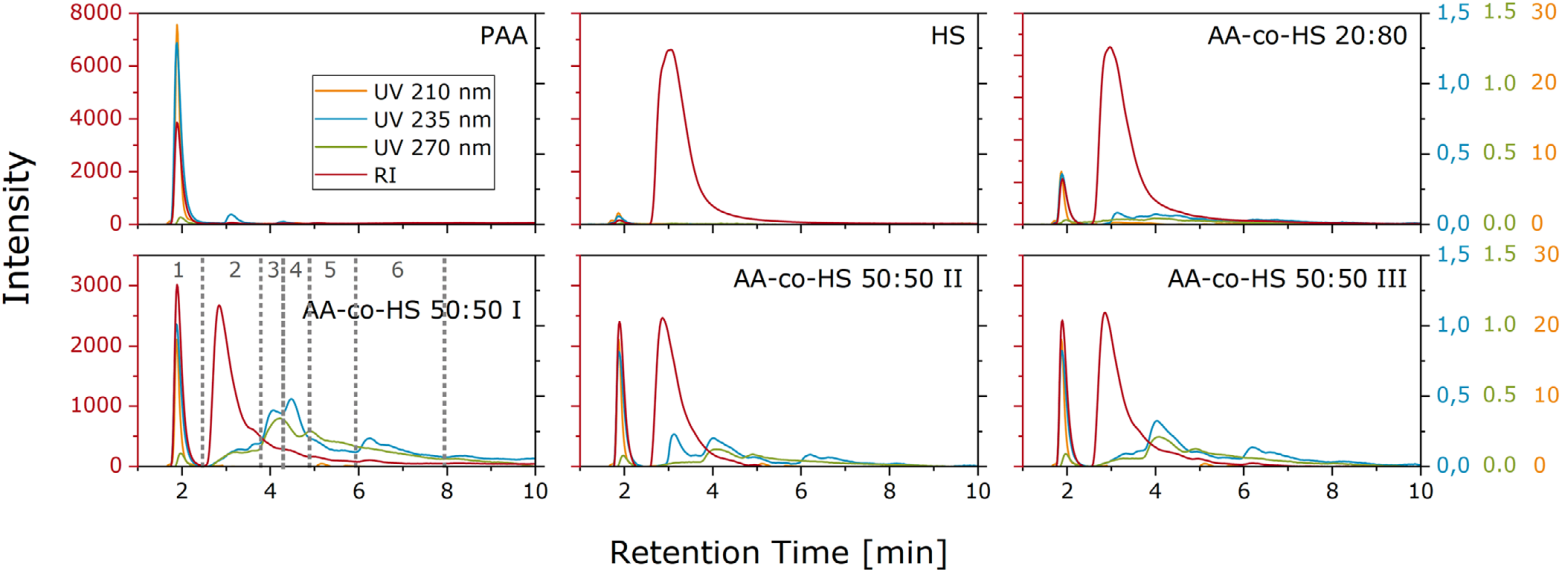
RI and UV chromatograms of all samples separated on a Jordigel DVB Polyamine column

Various stationary and mobile phases were tested for HPLC analysis. The best results were achieved with Jordigel DVB Polyamine. The separations were also tested at different pH values ranging from pH 2 to 9. The best results were obtained at pH 2. An organic modifier did not influence the separation significantly. It was found that for UV detection, measurements at 210 nm showed only signals for PAA. The trace at 235 nm showed signals for PAA and signals for what is believed to be the copolymer. The trace at 270 nm showed almost exclusively the signals of the latter, with negligible contribution from PAA. Measurements with even higher wavelengths showed less intensity and distortion. Figure 3 depicts the RI and UV chromatograms of all samples. The RI detector as a nonspecific concentration sensitive detector indicates all matter eluting from the column. As can be seen, a good separation between PAA and HS homopolymers was achieved. Determined by methods described in literature, it was found that PAA eluted in LCCC mode before HS, which eluted in LAC mode.^29^ The copolymer chromatograms also reflect the ratio of applied reactants in their RI signals. In the tailing of the HS side peak of the copolymer samples, several local maxima can be found that are not existent in the pure HS sample. Furthermore, at retention times between roughly 3 and 9 min, UV signals were observed that were not present in either homopolymer. These signals presumably result from the copolymer eluting in this retention time range.

### 3.3 Aqueous SEC measurements

**FIGURE 4:**
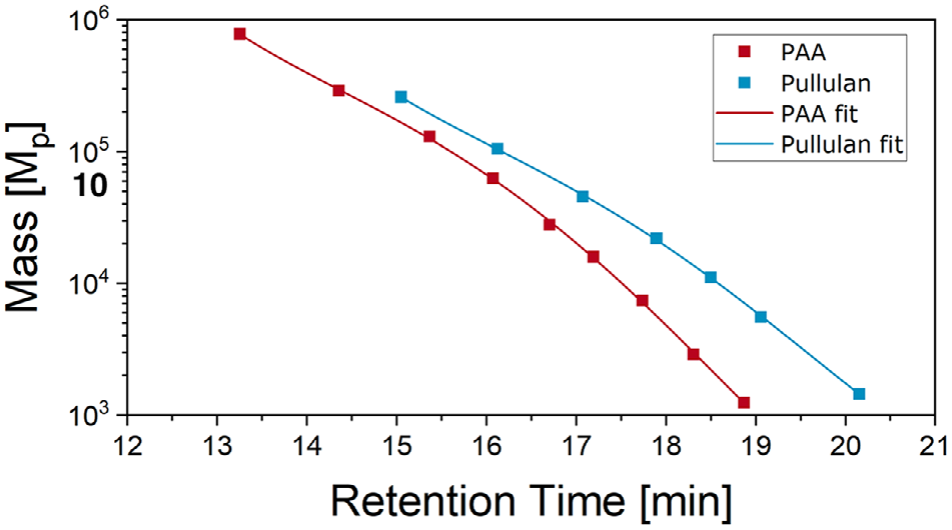
Calibration curves from pullulan and PAA standards for SEC analysis

The molar mass distributions of the samples were determined by aqueous size exclusion measurements. These measurements were carried at various pH values. The results at pH 2 were most meaningful. As the samples contain PAA and the polysaccharide HS, calibrations were obtained with PAA and Pullulan standards (Figure 4). Especially for lower masses, the retention times and correspondingly the calculated masses for both polymers differed significantly. We assume that the calculated molar mass distribution for a copolymer would not represent accurately the real values. As such, the calculated masses discussed below can considered only as virtual PAA or Pullulan related molar masses.

**FIGURE 5:**
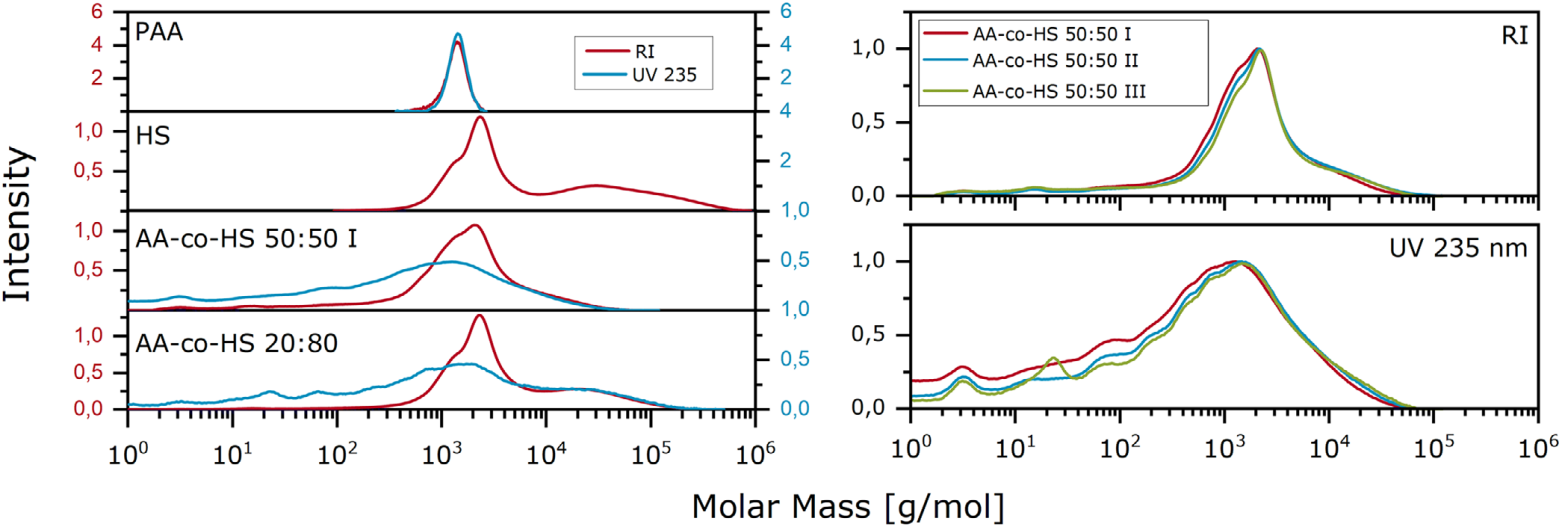
SEC chromatograms of homopolymers PAA 1250, Hydrolized starch, and copolymer samples

Nevertheless, SEC measurements shown in Figure 5 (left) are still representative of the overall mass distributions of the samples. For all measurements the PAA calibration was used. The PAA 1250 sample showed a narrow molar mass distribution (MMD) around 1400 g/mol. The HS sample showed a maximum intensity at about 2000 g/mol with a shoulder at 1500 g/mol and some higher masses up to 50 000 g/mol. In comparison, the copolymer samples showed a decrease of the higher molar masses.

This is possibly due to hydrolysis during the polymerization reaction. In contrast, the RI intensity for lower molar masses increased, suggesting lower degrees of polymerization for PAA and copolymer formed during the reaction. In the UV trace at 235 nm, the molar mass distributions of the copolymer samples extend to very low molar masses. This can be explained by short chains of PAA homopolymer or even unreacted AA. The distinct local maxima in UV molar mass distributions, however, could indicate the presence of copolymers.

A detailed comparison between the three copolymer samples comprising a 50:50 ratio between the two components, albeit with expectedly different PAA chain lengths, can be seen in Figure 5 (right). In the low molar mass range of the main distribution, the chain length of PAA and/or copolymer with the increase of sample numbers increases, as expected. We assume that most of the copolymer can be detected at the lower end of the distribution as there is no change in the high molar mass range of the distribution. Since the system is only calibrated for masses higher than 1000 g/mol, information on masses below that value are assumed to be unreliable. Here, the focus is on the comparison of the samples with each other, not the comparison of absolute masses. Slight adsorption effects could not be excluded completely.

**FIGURE 6:**
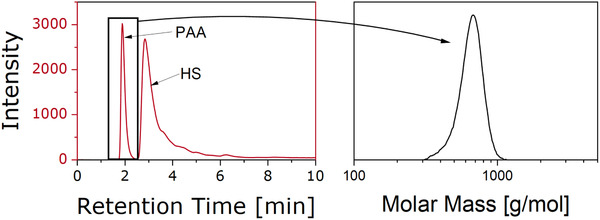
MMD characterization of the PAA homopolymer fraction formed during the reaction of AA‐co‐HS 50:50 I

One important piece of information to determine for the copolymer samples was the MMD of the PAA homopolymers within the samples. Since these were only formed during the reaction, their degree of polymerization was unknown. To characterize the MMD, the PAA fraction of the separation of AA‐co‐HS 50:50 I (shown in Figure 6) was further analyzed with size exclusion chromatography (SEC) (Figure 6, right). The calculated MMD was M_n _= 659 g/mol, M_w _= 681 g/mol, and M_p _= 680 g/mol. Although these values are somewhat unreliable due the aforementioned lack of calibration at masses below 1000 g/mol, they were in good agreement with the distributions observed in the ESI‐MS spectra (Figure 10: 5+6).

### 3.4 FT‐IR spectroscopy

**FIGURE 7:**
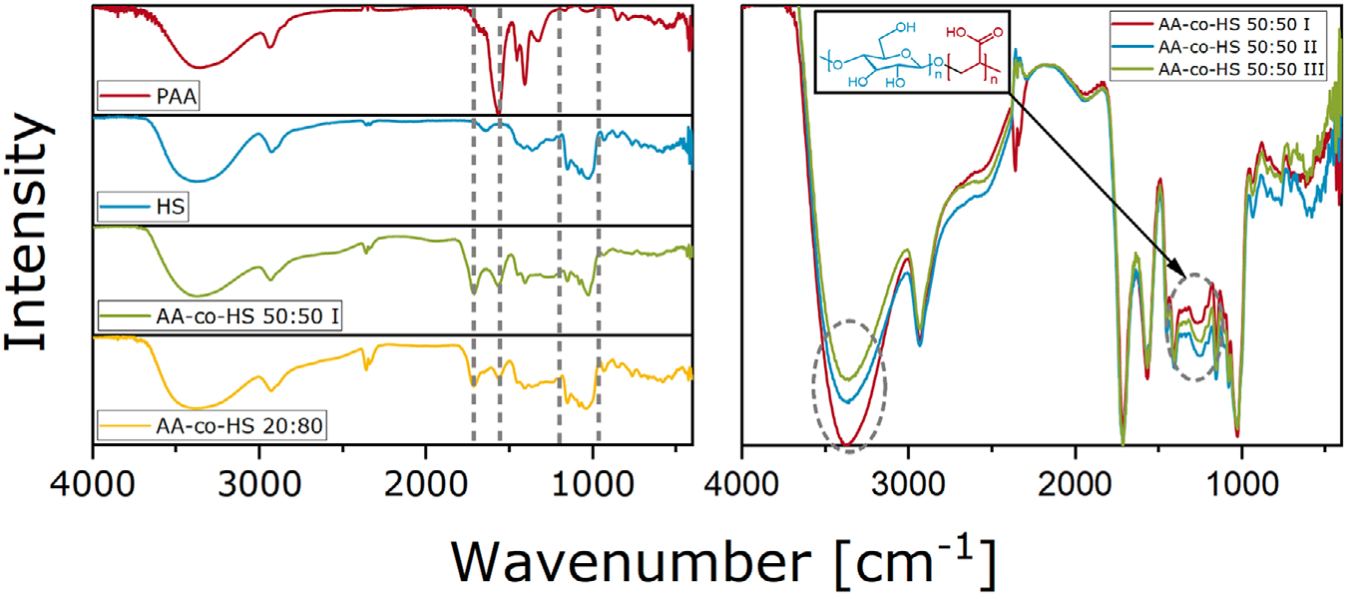
IR spectra of homopolymers and copolymer samples

To gain insight of the functional groups present in the samples, FT‐IR measurements were carried out. In Figure 7, FTIR spectra of the homopolymer and copolymer samples are displayed. Specific bands for the homopolymers are marked and can also be found in copolymer samples. Additionally, the copolymers show a signal at 1740 cm^−1^ that cannot be found in the homopolymers. The origin of this signal in the region for carbonyls is unclear.

In Figure 7 (right), all three samples with a ratio of 50:50 of AA:HS are compared. The OH‐signal at 3400 cm^−1^ decreases with the increase of the assigned sample number. This decrease can be attributed to fewer end groups and correspondingly longer PAA chains with increasing sample numbers (I‐III). The other notable difference in these spectra is located between 1400 and 1300 cm^−1^ and at about 900 cm^−1^. These signals are in the region of C‐O‐stretching bands and indicate the existence of the depicted copolymer structure in the samples. The difference between the samples can be interpreted as a preference toward the formation of ether bonds for longer PAA chains instead of C‐C bonds via free radical polymerization.

### 3.5 Fractionation

To further investigate the copolymer samples, some fractionations of AA‐co‐HS 50:50 I were prepared offline as highlighted in Figure 3.

**FIGURE 8:**
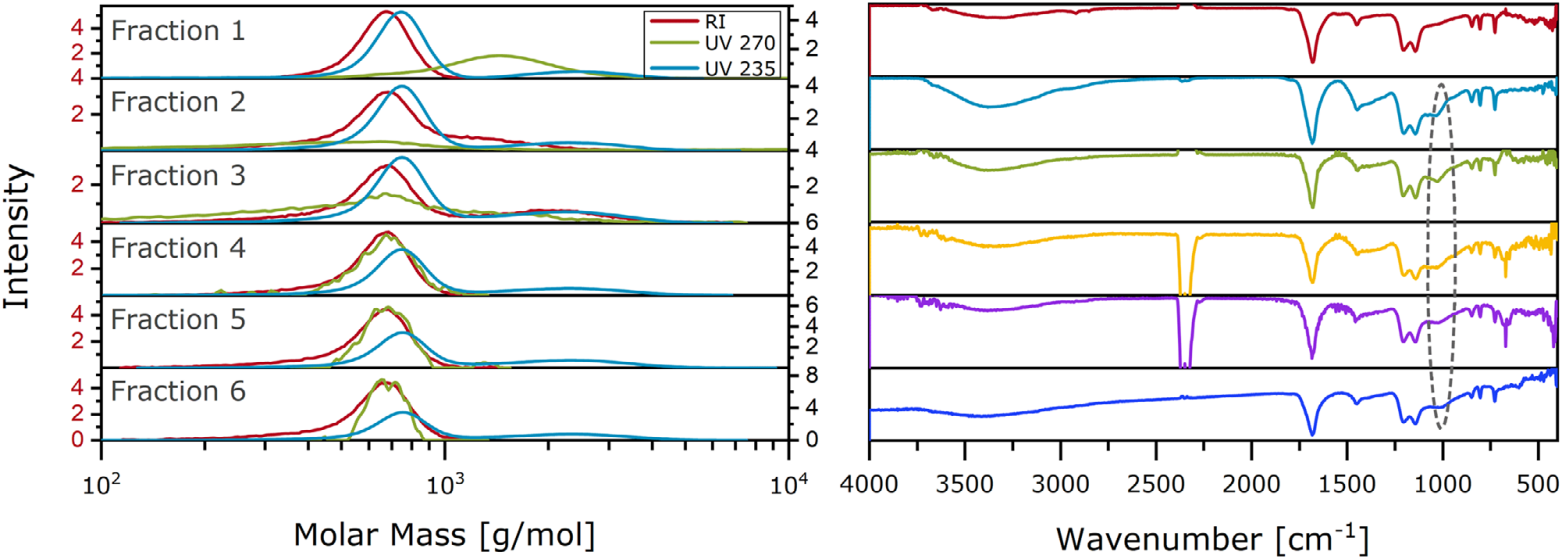
SEC chromatograms (left) and IR spectra (right) of the fractions of AA‐co‐HS 50:50 I as shown in Figure 3 bottom left

Based on the UV signals, six fractions were collected. Figure 8 depicts the SEC chromatograms (left) and FTIR spectra (right) of each fraction. The FTIR spectra did not show the specific signal at 1760 cm^−1^ for copolymer species as seen in Figure 7. Across the six fractions, spectra only varied in their signals at approximately 1000 cm^−1^. Again, this may be due to a change in the C‐O signal intensity. This band is only contained in fractions 2‐6, which were expected to contain the actual copolymer. The lack of this 1000 cm^−1^ band in fraction 1 is expected as it contained only the PAA homopolymer.

On the left in Figure 8, the molar mass distributions of all fractions are given. The RI signals show a mean molar mass at approximately 700 g/mol, with a second distribution at higher mass of 2500 g/mol in fractions 2 and 3. This distribution can also be observed in all fractions in the UV 235 distribution. While the lower molar mass distribution likely represents the PAA homopolymer, the higher molar mass distribution should represent the HS (fraction 2 and 3) and/or the copolymer. At UV 270 nm, which only shows signals from copolymers, a shift to lower molar masses can be seen with increasing fractions number. Also, an increase in intensity with increasing fraction number is obvious. These spectral changes suggest that the copolymer molar mass distribution must be between 600 and 2000 g/mol, considerably lower than the mean molar masses of the HS homopolymer. This may indicate a preference of lower mass HS to react with AA, or that hydrolysis takes place during the reaction.

### 3.6 LC on C18 phase

To complement the LC separation on the Jordigel DVB Polyamine column, additionally the YMC‐Triart C18 ExRS as a high‐coverage C18 phase was used. In addition to RI and UV detection (not shown), measurements were carried out with ESI‐MS and ELSD detection.

**FIGURE 9:**
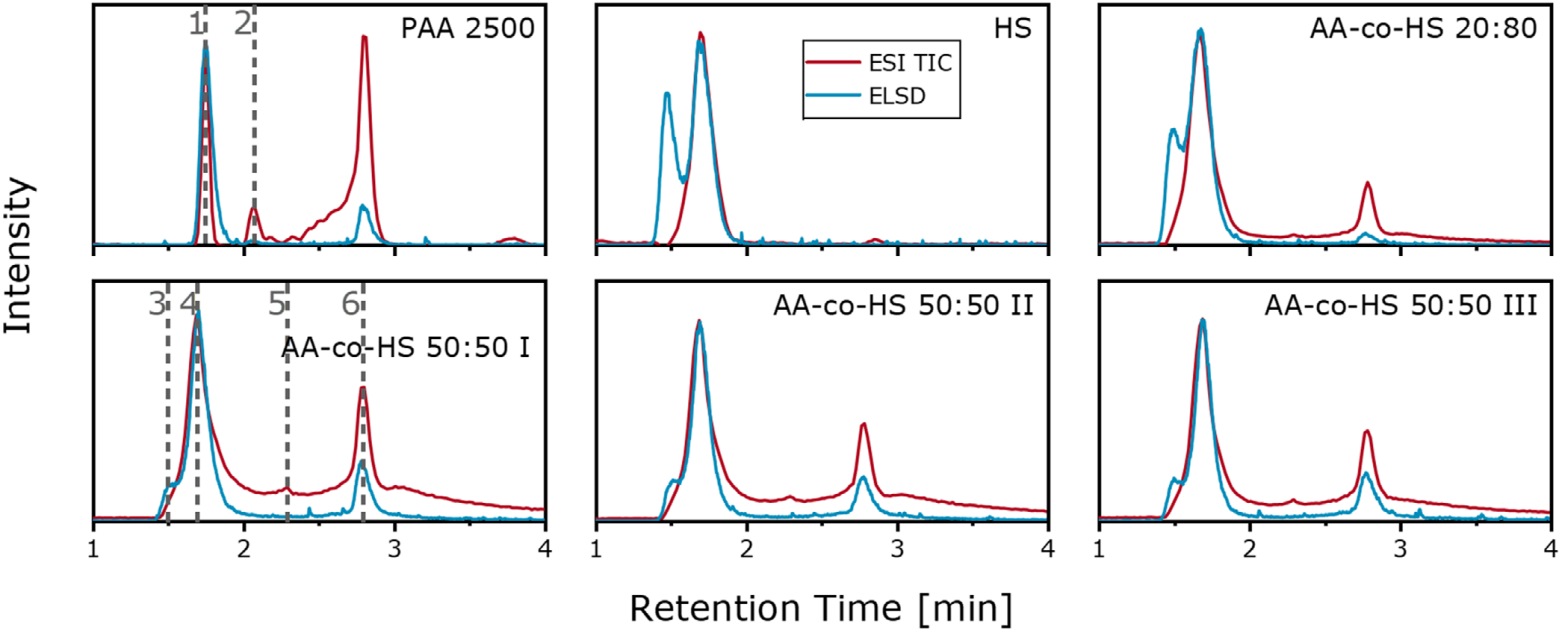
ELSD and ESI‐TIC chromatograms of all samples for separation on YMC‐Triart C18 ExRS column

Here, the elution order was reversed with respect to the previous measurements. HS elutes in LCCC mode, followed by elution of PAA in LAC mode.^29^ The total ion chromatograms and ELSD chromatograms of the samples are given in Figure 9.

**FIGURE 10:**
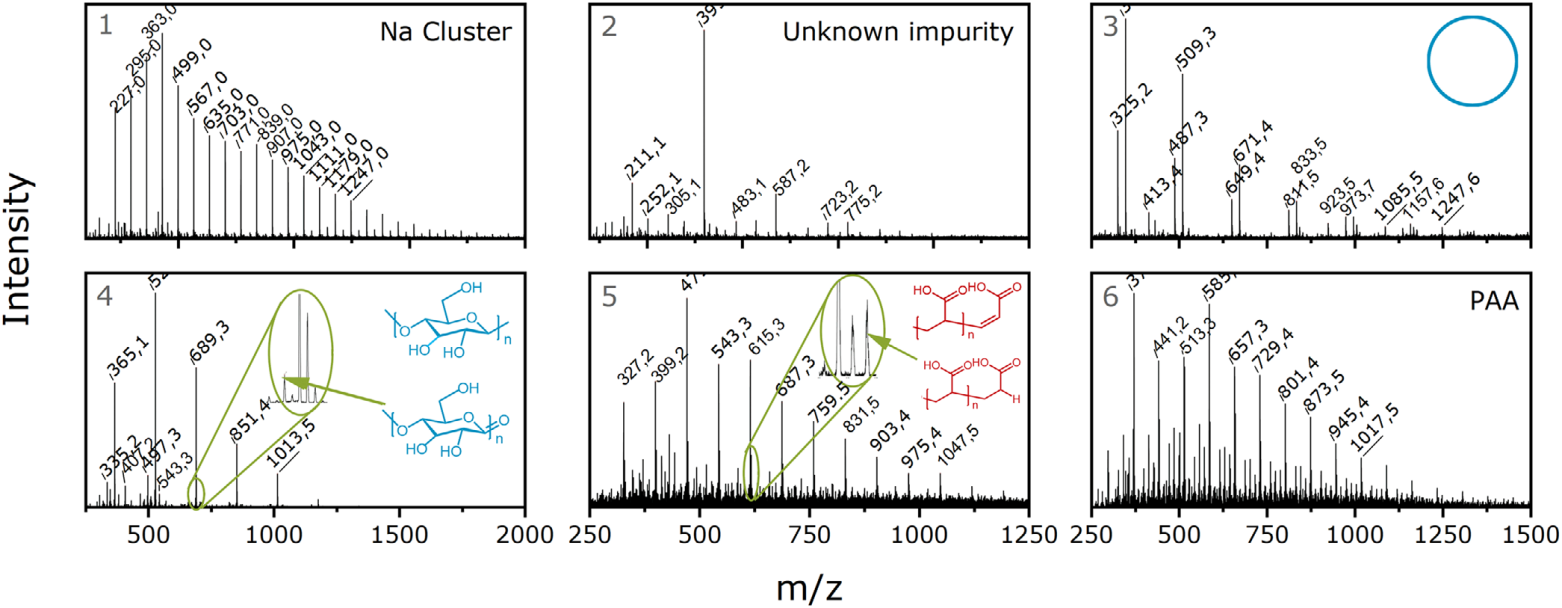
ESI‐MS spectra of the marked retention times in Figure 9

For HS, linear (1.6‐2.0 min) and cyclic (1.3‐1.6 min) polymers are visible. Comparison of ELSD and MS suggests that the cyclic polymers are not readily ionizable. Since PAA is present in the form of sodium salts, Na clusters in the form of [Na + x(sodium formiate)]^+^ with distances of 69 Da are present in the respected mass spectrum at 1.7 min, the same retention time as HS. During chromatography, some of the sodium ions from the PAA sodium salt were replaced with protons from formic acid. The remaining sodium and formate ions formed clusters. PAA homopolymer detected in MS in the form of [(M‐xNa+xH)+H]+ elutes at 2.5‐3.0 min in adsorption mode. An additional signal for PAA is located at 2.1 min and consists of an unknown impurity. For the copolymer samples, peaks of HS and PAA can be also identified. Additionally, a peak at 2.3 min is present. To clarify the structures in detail, six mass spectra from the location of the dotted lines in Figure 9 are shown in Figure 10.

For AA‐co‐HS 50:50 I different end groups for the homopolymers were found. For homopolymer HS, a cyclic form with no end groups (mass spectrum 3) was identified, alongside two series with other end groups (mass spectrum 4), either with high or with low intensity. The most probable structures are shown in the spectra. For PAA, one major and one minor distribution with different end groups can be seen in mass spectrum 5. In spectrum 6, a third distribution is shown, which could not be assigned to a specific end group. The most probable structures were derived from literature.^28^, ^30^ No copolymer structure was detected in any of the samples. This is most likely a result of the low amount of copolymer in the samples as well as the complexity of the spectra leading to a reduced sensitivity of MS for the copolymers. For ionization of PAA, it was crucial to inject a high sample amount into the mass spectrometer in combination with a high concentration of sodium trifluoroacetate as an ionization aid. Only at sufficiently high injection amounts did ionization of PAA take place. Therefore, the copolymers could not be ionized as their concentrations were too low.

### 3.7 NMR spectroscopy

**FIGURE 11:**
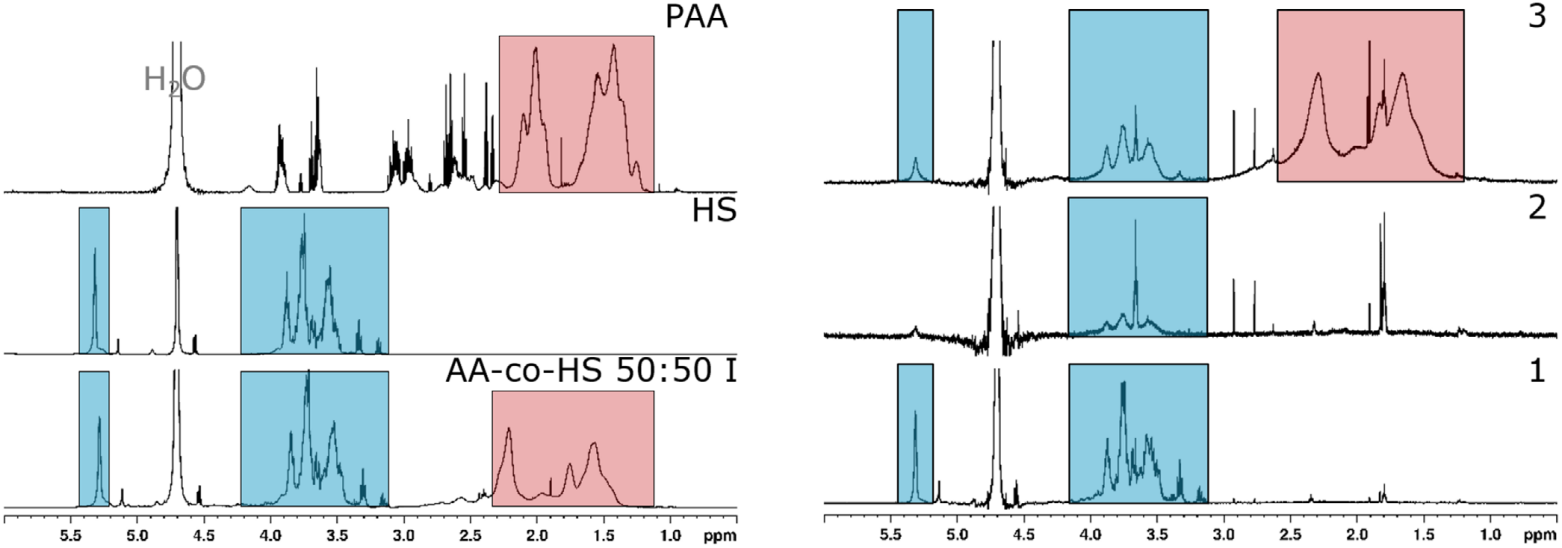
1H NMR spectra for homopolymers and AA‐co‐HS 50:50 I and the three fractions collected from chromatogram in Figure 12

To complement the results, NMR spectra were collected. In Figure 11, ^1^H‐NMR spectra of PAA, HS, and AA‐co‐HS 50:50 I are shown. Signal regions specific for both homopolymers are marked. For the copolymer sample, signals in the region of both homopolymers are present. The signals in the copolymer spectrum are, however, broader and less distinct. These features are indicative of the presence of copolymers.

**FIGURE 12:**
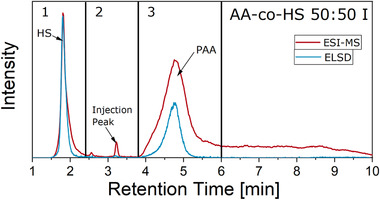
ELSD chromatogram and ESI‐TIC of AA‐co‐HS 50:50 I for gradient separation on YMC‐Triart C18 ExRS

The material collected in the above fractionation (see Section 3.5) was insufficient for NMR investigation. Coarser fractionation in three fractions was hence obtained. The elution from Figure 9 was improved by applying a gradient and the sample AA‐co‐HS 50:50 I was fractionated as shown in Figure 12.

The corresponding NMR spectra can be found in the right section of Figure 11. The existence of HS signals in fraction 3 is strong evidence of the presence of copolymers. Also, the broadening of the PAA and HS signals in this fraction compared to fraction 1 or pure homopolymers can be attributed to the formation of copolymers. The low intensity, broader signals of HS in fraction 2 are presumably the result of a high molar mass HS, consistent with SEC measurements in Figure 5.

^13^C‐NMR spectra of PAA, HS, and bulk copolymer (not shown) also support the same assumption derived from the IR‐measurements shown in Figure 7 regarding the presence of one copolymer structure in the samples.

### 3.8 2D chromatography

**FIGURE 13:**
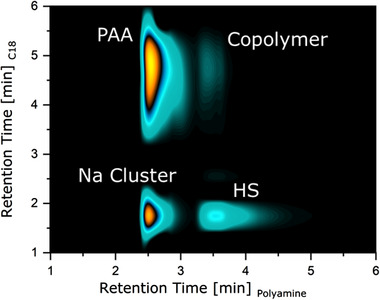
2D chromatogram of AA‐co‐HS 50:50 I with separation in Figure 12 as first (vertical) and separation in Figure 3 (bottom left) as second (horizontal) dimension

2D chromatography was conducted by combining the separation shown in Figure 12 as the fist (vertical) and the separation shown in Figure 3 as the second (horizontal) dimension. The ELSD chromatogram for AA‐co‐HS 50:50 I is shown in Figure 13.

In addition to the homopolymers, two additional peaks were found. One peak was attributed to sodium clusters. The presence of sodium was discovered previously only in the PAA homopolymer sample, but not in the copolymer samples. The reason for this is that the sodium cluster peak always coeluted with one of the other peaks in 1D separations. Sodium clusters are not distinguishable in UV and are poorly ionizable with ESI, as are the homopolymers. As such, these sodium clusters could not be seen before. The presence of sodium was verified by characterizing the fraction of the 2D chromatogram with ESI‐MS (not shown). Here, the same Na‐formate clusters as for the PAA samples were seen.

An additional peak at the upper right (Figure 13) can be attributed to the copolymer, since it appears as the second peak in both dimensions where the copolymer was expected. This peak was of only very low intensity. The ELSD vol% of copolymer for all samples were calculated to be 1.8% for AA‐co‐HS 50:50 I, 1.3% for AA‐co‐HS 50:50 II, 1.2% for AA‐co‐HS 50:50 III, and 0.4% for AA‐co‐HS 20:80. These results are in good agreement with the 1D UV chromatograms and NMR results. The trend of decreasing product yield with increasing PAA chain length as well as with an increasing amount of PAA is also in accordance to findings from others.^7^

## 4 CONCLUDING REMARKS

Within this work chromatographic conditions were developed that allow a near‐baseline separation of PAA and HS. All copolymer samples contained significant amounts of both PAA and HS homopolymers, making the detection of a low abundance copolymer a challenging task.

Two systems appeared promising for chromatography: the separation of PAA in SEC mode from HS in LAC mode (Jordigel DVB Polyamine), as well as the separation of HS in SEC mode from PAA in LAC mode (YMC‐Triart C18 ExRS). Though the copolymers could not be separated from homopolymers entirely, a baseline separation of homopolymers was achieved for both chromatographic separations. The copolymer did not elute between both homopolymers, as would have been assumed. Instead, the copolymer eluted within the second homopolymer peak. The challenge of chromatographic separation was associated with the low amount of copolymer present, in combination with a broad distribution in molar mass and microstructure. The combination of RI and UV detection gave additional insight into the copolymer composition. SEC experiments enabled comparative determination of the molar mass distributions of copolymers. In addition, FTIR investigations clearly showed different spectra for copolymers as compared to the homopolymers, thereby confirming the successful formation of copolymer linkage groups. In addition, the formation of a copolymer via the formation of ether bonds was indicated, although the complete mechanism could not be elucidated in this study. The combination of LC and mass spectrometry led to detailed information on the structures and functional end groups. The copolymers were not detectable with ESI‐MS. However, the existence of several different end groups in HS, as well as PAA was confirmed by ESI‐MS, offer further mechanistic insight into the copolymerization reaction and a target for further mechanistic studies. Additional evidence of the copolymer formation was gained from NMR measurements. Finally, 2D chromatography led to the separation of the copolymers from both homopolymers as well as the additional separation of sodium clusters. The amounts of copolymer were calculated to less than 2% by ELSD vol% for each sample.

The methods described in this work provide a powerful toolset to characterize copolymerization products of HS and PAA. They open the possibility to correlate the physicochemical properties of such complex mixtures with their actual composition and adjust optimize it as desired.

## CONFLICT OF INTEREST

The authors declare no conflicts of interests.
